# Gut Microbiome Associates With Lipid-Lowering Effect of Rosuvastatin *in Vivo*

**DOI:** 10.3389/fmicb.2018.00530

**Published:** 2018-03-22

**Authors:** Yinhui Liu, Xiaobo Song, Huimin Zhou, Xue Zhou, Yunlong Xia, Xin Dong, Wei Zhong, Shaoying Tang, Lili Wang, Shu Wen, Jing Xiao, Li Tang

**Affiliations:** ^1^Department of Microecology, College of Basic Medical Sciences, Dalian Medical University, Dalian, China; ^2^Department of Medical Biology, Faculty of Health Sciences, University of Tromsø, Tromsø, Norway; ^3^Department of Microbiology, College of Basic Medical Sciences, Dalian Medical University, Dalian, China; ^4^Department of Clinical Laboratory, The Second Hospital of Jiaxing, Jiaxing, China; ^5^Department of Cardiology, The First Affiliated Hospital of Dalian Medical University, Dalian, China; ^6^Department of Oral Pathology, College of Stomatology, Dalian Medical University, Dalian, China

**Keywords:** gut microbiome, 16S rRNA sequencing, rosuvastatin, hyperlipidemia, hypolipidemic effect

## Abstract

**Background:** Statin has been widely used to treat hyperlipidemia because of its high potency in decreasing cholesterol levels. The present study aimed to examine the lipid-lowering effect of rosuvastatin and the composition, diversity and species abundance of gut microbiome in association with rosuvastatin efficacy. Trial registration: ChiCTR-ORC-17013212 at the First Affiliated Hospital of Dalian Medical University, November 2, 2017.

**Results:** Totally 64 patients with hyperlipidemia were treated with 10 mg/day of rosuvastatin for 4–8 weeks. Blood lipid indicators triglycerides (TG), total cholesterol (TC), high density lipoprotein (HDL), low-density lipoprotein cholesterol (LDL-C) were measured before and after the treatment. Stool samples were collected right after the treatment. Following total DNA extraction and PCR amplification of 16S rRNA gene, Illumina sequencing was performed for gut microbiome identification, classification and characterization. All the patients showed a significant blood lipid reduction after the treatment. The patients were grouped according to parallel manner design. Group I had 33 patients whose blood lipid levels dropped to the normal levels from week 4, with 58.5% reduction in LDL-C and 26.6% reduction in TC. Group II had 31 patients whose blood lipid levels were still above the normal levels after 8 weeks therapy, but with 41.9% reduction in LDL-C and 31.2% reduction in TC. Based on Operational Taxonomic Unit data, Alpha-diversity by Shannon Index was different between the two groups, and beta-diversity by Principle Component Analysis exhibited separated patterns of the two groups. The differences were also observed in the relative-abundance at phylum, family, and genus levels of the two groups. Linear discriminate analysis illustrated that the abundance of 29 taxa was higher in group I, while the abundance of other 13 taxa was higher in group II. Phyla Firmicutes and Fusobacteria had negative correlation to LDL-C level, but Cyanobacteria and Lentisphaerae had a positive correlation to LDL-C level. Moreover, gender and age were also found somehow correlated to microbial community composition.

**Conclusion:** Rosuvastatin therapy had different blood lipid-lowering effect on hyperlipidemia. The gut microbiota exhibited variation in community composition, diversity and taxa in association to rosuvastatin hypolipidemic effect. These results indicate that modulation of gut microflora, especially the negative/positive correlated species might strengthen statin efficacy in statin-inadequate patients.

## Introduction

Hyperlipidemia, high blood cholesterol and Triglycerides, is considered a major risk factor for coronary heart disease, ischemic stroke, and peripheral artery disease. The causes of hyperlipidemia can be primary such as genetic defects or secondary such as excessive eating, high saturated fatty acid diet, smoking, less physical activity, obesity, certain medications. Due to the changes in lifestyle and dietary, blood lipid levels and the incidence of cardiovascular diseases increase greatly in Chinese people lately (Pisciotta et al., 2015). According to WHO Representative Office in China, annual cardiovascular events are predicted to increase by 50% between 2010 and 2030 considering only population aging and growth in China, but by 23% in addition when considering the tendency in blood pressure, TC, diabetes, and active smoking etc. Overall an increase of approximate 21.3 million cardiovascular events and 7.7 million cardiovascular deaths pose a serious challenge to Chinese public health-care system.

To date, statin drugs are widely used for treatment of hyperlipidemia. The action mechanisms of statins include reducing mevalonic acid synthesis through inhibiting HMG-CoA reductase, elevating the plasma expression of LDL-C receptor so as to promote LDL-C uptake, decreasing the synthesis of hepatic VLDL-C and LDL-C, and accelerating the clearance of plasma LDL-C. Rosuvastatin is the third-generation of statin drugs. It acts as a synthetic HMG-CoA reductase inhibitor made up of amino-pyrimidine derivatives. Water-soluble and lipid-soluble rosuvastatin enters the human body without metabolism, thus enhance its lipid-lowering activity. Moreover it reduces not only cholesterol levels but also other lipid profiles such as TGs. Rosuvastatin is therefore widely used in primary and secondary prevention of coronary artery disease (Athyros et al., 2002; Zhou and Liao, 2010; Mihaylova et al., 2012; Perk et al., 2012; Taylor et al., 2013; Al-Mohaissen et al., 2016; Lamprecht et al., 2017; Yeramaneni et al., 2017). However, it has been noticed since 1990s that about 20% patients showed inadequate or resistant response to statin therapy regardless of increasing doses (Wang, 2011; Chan et al., 2016). The high dose of statin uses tends to induce the adverse effects such as muscle toxicity and hepatic injury.

Gut microflora are essential in human metabolism of dietary components and host-generated substances, particularly lipid metabolism. The total amount of gastrointestinal bacteria is around 10^14^ of a healthy adult, more than the total number of body cells (Simren et al., 2013), which comprise a complex and metabolic active ecosystem (Parkes et al., 2008). The quantity and diversity of gut microbiota affect the development of some chronic diseases (Balsari et al., 1982; West, 2014; Erdman, 2016; Knip and Siljander, 2016) through involving in both nutrient and energy metabolism and host immune defense (Heyman et al., 1989; Martin et al., 2007; Ley et al., 2008). Recent studies reveal a close relationship between the gut microbiota and hyperlipidemia. Hyperlipidemia patients are often accompanied with intestinal flora disorders that might further impair lipid metabolism. In fecal flora of hyperlipidemia patients, Gram-negative bacillus species increase, as well as the bacteria producing SCFAs reduced significantly (Wang et al., 2016). Dong and coworkers reported a change in composition of intestinal flora and its impact on lipid-lowering effect in obese rats (Dong et al., 2016).

In order to understand the association between the statin effect and gut microbiota as well as the potential treatment for statin inadequate patients, the present study aimed to examine (1) the lipid-lowering effect of rosuvastatin on hyperlipidemia, (2) the fecal microbiota composition, diversity and abundance at phylum, family and genus levels, and (3) gut microbiota variation on age and gender in association to rosuvastatin effectiveness.

## Materials and Methods

### Subjects and Study Design

The study was performed with the approval of the Ethics Committee of The First Affiliated Hospital, Dalian Medical University, China (No. YJ-KY-FB-2015-12). All study participants provided with written informed consent before their enrolment. We consecutively enrolled 64 subjects with primary hyperlipidemia (55–75 years, 45 men and 19 women) at the First Affiliated Hospital of Dalian Medical University from December 2015 to October 2016. The diagnosis of hyperlipidemia was based on the following inclusion criteria set by the Chinese society of Cardiology in 2007: serum LDL-C ≥ 3.64 mmol/L, HDL ≤ 3.64 mmol/L, TG ≥ 1.70 mmol/L, TC ≥ 5.72 mmol/L. Patients were excluded if they had at least one of the following conditions: (1) a history of antibiotic application (within 3 months); (2) long-term use of steroid hormone, thyroid hormone, contraceptive drugs and drugs that affect lipid parameters of serum; (3) active liver and kidney diseases; (4) uncontrolled hypothyroidism; (5) adrenocortical hyperfunction; (6) acute and chronic disorders of the digestive system. All patients received 10 mg/day of rosuvastatin for 4–8 weeks and the levels of serum lipids including LDL-C, HDL, TG, and TC were measured pre- and post-treatment using Assay Kits (BioSino Bio-Technology & Science Inc., Beijing, China). Parallel manner design was applied as Group I with 33 patients whose blood lipid levels dropped to the normal levels from week 4 and Group II with 31 patients whose blood lipid levels were still above the normal levels after 8 weeks rosuvastatin intervention. Fresh fecal specimens were collected from each subject with a sample box sterilized by ethylene oxide within 30 min of defecation and then stored at -80°C for downstream analysis.

### Isolation of Total DNA From Fecal Samples

Genomic DNA was extracted from the fecal sample (50–100 mg) with an E.Z.N.A. Stool DNA Kit (Omega Bio-Tek, Inc., Norcross, GA, United States) according to the manufacturer’s protocol. The total DNA quantitation was determined using Qubit V2.0 fluorometer (Invitrogen, Carlsbad, CA, United States) and the quality of DNA was evaluated by agarose gel electrophoresis. Extracted DNA samples were stored at -80°C until further processing.

### 16S rRNA Gene Sequencing

16S rRNA gene sequences were amplified from the bulk DNA with the barcoded primers 341F (5′-CCTAYGGGRBGCASCAG-3′) and 806R (5′-GGACTACNNGGGTATCTAAT-3′), which covers V3–V4 regions of the 16S rRNA gene (Sundberg et al., 2013). After an initial denaturation step at 98°C for 1 min, PCR amplification was carried out using 30 cycles of 98°C for 10 s, 50°C for 30 s and 72°C for 30 s, and a final extension at 72°C for 5 min. PCR reactions were performed on an ABI GeneAmp 9700 PCR system (Applied Biosystems, Foster City, CA, United States). The 16S amplicons were purified with GeneJET PCR Purification Kit (Thermo Fisher Scientific, Waltham, MA, United States) and DNA library was constructed by using TruSeq DNA PCR-Free Library Kit (Illumina Inc., San Diego, CA, United States). The libraries were then sequenced by Hiseq sequencing System (Illumina Inc., San Diego, CA, United States). After trimming barcodes and primer sequences, the remaining sequential reads of each sample were linked by FLASH V1.2.7 (Magoc and Salzberg, 2011) (Fast Length Adjustment of SHort reads^1^). Quality control of the sequence data was done using the open-source software QIIME (Caporaso et al., 2010) (Quantitative Insights Into Microbial Ecology, version 1.7.0^2^). The sequences were removed according to the following criteria: read length no longer than 150 bp, average quality score less than 20 and homopolymers longer than eight nucleotides. Sequences identified as chimeras were filtered from further study with UCHIME^3^ (Edgar et al., 2011), using the GOLD reference database^4^ (Haas et al., 2011).

### Taxonomic Classification of 16S Gene Sequences

Taxonomy-based analyses were conducted using the Ribosomal Database Project Naive Bayes classifier (Cole et al., 2005), with a confidence of 95%. The remaining sequences with more than 97% similarity threshold were clustered into OTUs. Each read was classified to phylum, class, order, family, and genus with the SILVA reference database. The relative abundance of species was calculated by dividing the number of sequences of each taxonomic level by the total number of sequences per sample.

### Bioinformatic and Statistical Analysis

Microbiome diversity was estimated by alpha diversity (within communities) and beta diversity (between microbial communities) analyses. Alpha diversity evaluated species richness with ACE index and Chao 1 index, diversity with Shannon and Simpson indexes, and Coverage index (Human Microbiome Project Consortium, 2012). The number of OTUs also reflects the richness of microbiome. OTU was assigned by 3% divergence cutoff value. Generation of rarefaction curves and calculation of alpha diversity indices were done with Mothur software v1.20.0 program (Schloss et al., 2009). PCA and the ANOSIM were performed to evaluate differences between microbial communities. The phylogenetic distance was calculated and plotted using UniFrac distance metric between groups. The correlation between microbiome taxa and rosuvastatin effectiveness were assessed using LDA effect size (LEfSe) at various taxonomic ranks (Segata et al., 2011). The LDA score of greater than 3.0 is thought to be significant by default. LEfSe data were analyzed using statistical software R (version 3.2.3 ^5^), *Q*-values were adjusted using the Benjamini–Hochberg FDR correction. The results were shown on taxonomy bar-chart and cladogram plots. Student’s *T*-test was used to identify the differences between two groups, with *P* < 0.05 being considered a significant difference. To analyze the relationships of fecal microbiome with rosuvastatin effectiveness, Spearman correlations were calculated pairwise and a heatmap based on Spearman correlation coefficients was plotted (Azen and Sass, 2008). *R*-value of Spearman Correlation coefficient represented the relevance of two group, *r* < 0 was considered as a negative correlation, as well as *r* > 0 was a positive correlation. *P*-values were considered significant using a FDR of 25%.

## Results

### Efficacy of Rosuvastatin

A total of 64 patients with primary hyperlipidemia received rosuvastatin treatment at the First Affiliated Hospital of Dalian Medical University. The therapy was well tolerated in all the subjects with good compliance and with no obvious drug-related AEs during rosuvastatin administration. The lipid patterns were without difference at baseline. The changes of serum lipids were determined after rosuvastatin administration for 4–8 weeks. TC, TG, and LDL-C reduced significantly compared to baseline, as well as HDL elevated slightly in all subjects. According to the degrees of lipid-lowering, subjects were classified into two parallel groups: group I, with high efficacy of rosuvastatin treatment; group II, with relatively low efficacy of rosuvastatin treatment. The patients of group I achieved apparently a greater decrease in TC and LDL-C. TC was -41.9% in group I and -26.6% in group II, LDL-C was -58.5 and -26.6% in two groups, respectively. However, the decline of TG level was not significant between the two groups. A summary of serum lipids before and after rosuvastatin treatment was presented in **Table 1**.

**Table 1:** Lipid-lowering effect of rosuvastatin treatment on 64 hyperlipidemia patients.

Patient group#	Number (male, female)	Age (mean)	Triglycerides (mmole/L) (mean ±*SD*)		Cholesterol (mmole/L) (mean ±*SD*)					
					Total cholesterol		Low-density lipoprotein		High density lipoprotein	
			Pre-treatment	Post-treatment	Pre-treatment	Post-treatment	Pre-treatment	Post-treatment	Pre-treatment	Post-treatment
Group I	33 (23,10)	64.24	3.56 ± 1.93	1.29 ± 0.55	5.36 ± 1.63	3.12 ± 0.51^∗^	1.15 ± 0.27	1.16 ± 0.36	3.52 ± 1.02	1.46 ± 1.27^∗^
Group II	31 (22,9)	65.55	3.13 ± 1.57	1.50 ± 0.66	5.85 ± 1.36	4.26 ± 0.86	1.19 ± 0.32	1.21 ± 0.26	3.65 ± 0.93	2.50 ± 0.70

*^#^All patients were classified into two groups: Group I patients’ blood lipid levels back to the normal after rosuvastatin treatment, Group II patients’ blood lipid levels above the normal after treatment. ^∗^Significant difference between pre- and post-treatment lipid levels measured with Assay Kits (BioSino Bio-Technology & Science Inc., Beijing, China), P < 0.05*.

### Analyses of Alpha and Beta Diversity

**FIGURE 1:**
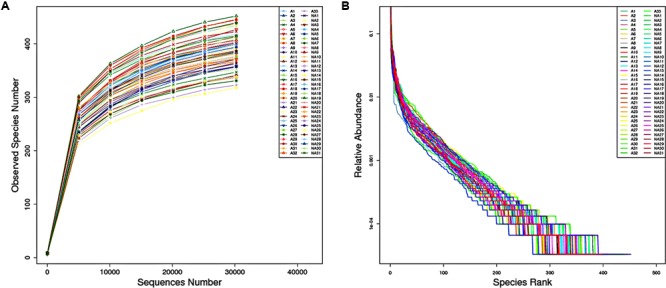
**(A)** Rarefaction curve for the number of operational taxonomic units (OTUs) observed in individual sample against sequences number. **(B)** Rank abundance curve. The horizontal axis shows the OTU abundance in rank order; the vertical axis shows the relative abundance of sequences on a logarithmic scale.

To compare the microbial diversity between groups I and II, the extracted genomic DNA from stool samples were subjected to 16S rRNA gene sequencing. Rarefaction curve and rank abundance curve were plotted to analyze the ecological parameters such as diversity or richness which reflected the depth and coverage of sequencing. As seen in **Figure 1A**, a rarefaction curve of the observed species number versus sequences in each sample showed the efficiency of the sequencing and sufficient sampling depth, as well as high similarity among group members. In **Figure 1B**, the line slopes of rank abundance curve indicated species (OTU) evenness, line lengths reflected OTU richness, and the long tail represented less abundant taxa. Based on the OTU data, alpha diversity measurement was shown on several indices. The ACE index and Chao 1 index were used to explore the total number of species in an ecosystem, and were calculated through two different formulas. The Shannon index and Simpson index reflected the heterogeneity in the microbiome. The results revealed that richness indices, ACE and Chao 1, were not different comparing the samples from the two groups. However, a significant difference in alpha diversity was spotted by Shannon index (*P* < 0.05) of the two groups’ gut microbiome. Shannon indexes of groups I and II were 6.240.54 and 5.810.49, respectively, suggesting that the complexity of fecal microbiome could be positively correlated with rosuvastatin effectiveness. Furthermore, Coverage index of two groups were all more than 99.8%, suggesting a high sequencing coverage of samples in the trial.

**FIGURE 2:**
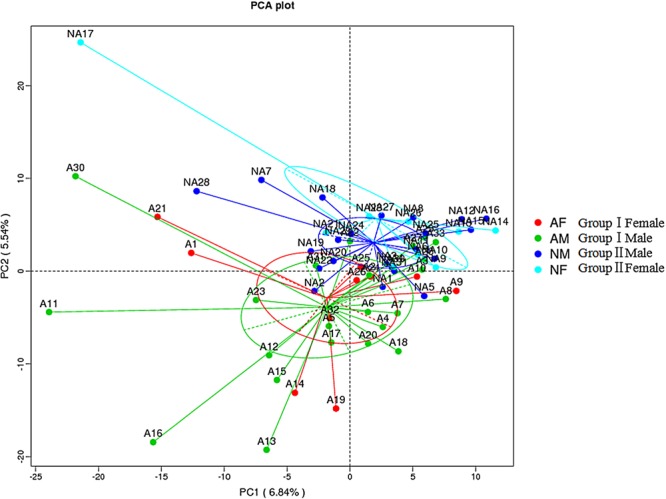
Beta diversity assessment of gut microbiome. Principles Component Analysis (PCA) was used and a scatterplot was generated to determine the phylogenetic distances between bacterial communities. The axes indicate the percentages of variation in the data for the bacterial communities. Dot symbols in different representing color that cluster together are circled in red, green, dark blue, and light blue, respectively.

We next performed beta diversity analysis to compare the similarity among gut microbiome of the two groups (**Figure 2**). PCA plot revealed that the gut microbiome of different groups was well separated from each other, microbial community of group I clustered in green circle, and those of group II gathered in blue circle. However, the community of male subjects was not readily differentiated from females within the group.

### Comparison of Gut Microbiome at Phylum, Family, and Genus Levels

**FIGURE 3:**
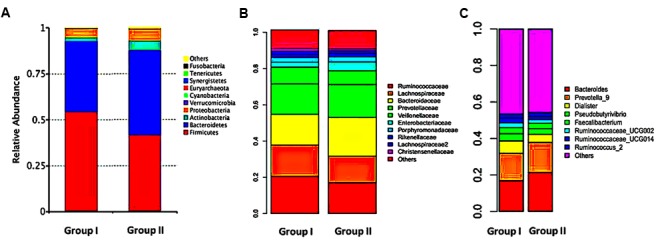
Relative abundance of gut microbiome. Gut microflora were profiled with the mothur pipeline. Phylum **(A)**, family **(B)**, and genus **(C)** level taxonomy are presented as percentage of total sequences. Different colored bars indicate different gut microbial taxa.

The relative abundances of overall microbiome between the groups differed at the phylum, family and genus levels, but most of these differences were without statistically significant. At the phylum level, sequences from the Firmicutes and Bacteroidetes phyla were most abundant in both the groups, accounting for 54.71 and 45.27% of the microbiome in group I and 38.38 and 44.75% in group II. Proteobacteria, Actinobacteria and Verrucomicrobia together comprised 6.69% in group I and 19.7% in group II. The relative abundance of Firmicutes, Verrucomicrobia, Tenericutes, and Fusobacteria were significantly increased in group I compared to those of group II, whereas group II exhibited a significantly higher bacterial abundance of Bacteroidetes, Actinobacteria, Cyanobacteria, and Lentisphaerae compared to group I (**Figure 3A**).

At the family level, the samples of group I were dominated by four families: Ruminococcaceae (20.61%), Lachnospiraceae (17.27%), Bacteroidaceae (16.89%), and Prevotellaceae (16.73%), but in group II were Bacteroidaceae (21.33%), Prevotellaceae (18.19%), Ruminococcacea (17.04%), and Lachnospiraceae (14.69%). Compared with group II, reads of group I showed obviously lower relative abundances of Bacteroidaceae, Prevotellaceae, Pseudomonadaceae, Rikenellaceae and higher relative abundances of Ruminococcaceae, Lachnospiraceae, Veillonellaceae, Christensenellaceae, Lactobacillaceae, Verrucomicrobiaceae, Coriobacteriaceae, and Clostridiaceae_1 (**Figure 3B**).

Constitutionals bacteria at genus level revealed that *Bacteroides* (16.93%), *Prevotella_9* (15.07%), *Dialister* (6.70%), *Pseudobutyrivibrio* (3.98%), *Faecalibacterium* (3.32%), *Ruminococcaceae_UCG-002* (2.66%), *Ruminococcaceae_UCG-014* (2.64%), and *Ruminococcus_2* (2.12%) were the dominant in group I, whereas the top genera in group II were, in turn, *Bacteroides* (21.35%), *Prevotella_9* (16.53%), *Dialister* (4.42%), *Pseudobutyrivibrio* (3.12%), *Faecalibacterium* (2.99%), *Bifidobacterium* (2.59%), *Parabacteroides* (2.40%), and *Escherichia–Shigella* (2.32%). Relative abundances at genus level differed apparently between two groups. Several genera of group I were significantly decreased than those in group II, including *Bacteroides, Parabacteroides*, and *Veillonella*. On contrast, 16 genera were elevated in group I, indicating there was a relationship between different microbiome compositions of gut microbiota and therapeutic effects of rosuvastatin (**Figure 3C**).

Composition and abundance of the gut microbiome were analyzed between the groups based on gender and age. The females of group I showed higher relative abundance of Firmicutes, Tenericutes (phylum level), and Ruminococcaceae, Lachnospiraceae, Coriobacteriaceae, and Alcaligenaceae (family level) compared to those of group II. On the other hand, the males of group I exhibited increased OTUs of Firmicutes, Tenericutes, Clostridiaceae_1, and declined OTUs of Bacteroidaceae compared to those of group II (**Table 2**). We also observed that distribution of gut microbiome was different on age. Relative abundance of Firmicutes, Euryarchaeota, Lachnospiraceae, Erysipelotrichaceae, Desulfovibrionaceae, Methanobacteriaceae, and Clostridiales_vadinBB60_group were higher in subjects aged from 55 to 65 of group I than those of group II, while lower abundance of Bacteroidetes, Actinobacteria, and Cyanobacteria were found in this subject community than those in group II. Patients aged 65–75 years of group I have significantly more abundant reads of Fusobacteria and notable lower OTUs of Bacteroidaceae and Peptostreptococcaceae as compared to those of group II. These data showed the distribution profiles of gut microbiome in rosuvastatin-treated patients with different age and clinical outcomes (**Table 3**).

**Table 2:** Relative abundance of gut microbiome taxa in according to gender and rosuvastatin effectiveness.

	Female		Male	
	Group I	Group II	Group I	Group II
Firmicutes	54.50	41.91^*^	54.71	46.61^*^
Bacteroidetes	38.39	46.00	38.47	44.28
Actinobacteria	2.10	5.21	1.52	2.11
Proteobacteria	4.66	6.19	4.91	6.72
Verrucomicrobia	0.10	0.15	0.20	0.12
Euryarchaeota	0.08	0.13	0.03	0.05
Synergistetes	0.03	0.12	0.02	0.02
Tenericutes	0.11	0.05^*^	0.09	0.07^*^
Prevotellaceae	16.62	20.50	16.81	17.26
Veillonellaceae	8.90	8.24	9.49	7.39
Bacteroidaceae	16.62	20.32	17.06	21.77^*^
Bifidobacteriaceae	1.20	4.99	0.75	1.60
Ruminococcaceae	21.46	14.49^*^	20.41	18.18
Lachnospiraceae	17.49	13.79^*^	17.08	15.02
Enterobacteriaceae	2.66	5.04	2.78	4.64
Porphyromonadaceae	2.50	2.97	2.71	2.81
Christensenellaceae	1.28	1.29	1.53	0.90
Pasteurellaceae	0.43	0.34	0.75	0.42
Erysipelotrichaceae	0.55	0.34	0.79	0.32
Rikenellaceae	1.91	1.92	1.66	1.81
Bacteroidales_S24-7_group	0.72	0.23	0.22	0.61
Streptococcaceae	0.67	0.72	0.84	0.93
Coriobacteriaceae	0.87	0.21^*^	0.73	0.48
Lachnospiraceae	1.67	1.15	1.62	1.72
Verrucomicrobiaceae	0.10	0.20	0.12	0.15
Clostridiaceae_1	0.52	0.80	0.44	0.35^*^
Desulfovibrionaceae	0.66	0.55	0.71	0.35
Alcaligenaceae	0.72	0.67	0.79	0.37

*^∗^Significant difference between the two groups, P < 0.05.*

**Table 3:** Relative abundance of gut microbiome taxa in according to age and rosuvastatin effectiveness.

	55–65 years		65–75 years	
	Group I	Group II	Group I	Group II
Firmicutes	0.5665	0.4491^∗^	0.5193	0.4570
Bacteroidetes	0.3716	0.4691^∗^	0.4020	0.4183
Actinobacteria	0.0166	0.0257^∗^	0.0173	0.0362
Proteobacteria	0.0428	0.0538	0.0560	0.0820
Verrucomicrobia	0.0006	0.0003	0.0032	0.0026
Cyanobacteria	0.0000	0.0011^∗^	0.0001	0.0001
Euryarchaeota	0.0004	0.0000^∗^	0.0005	0.0017
Prevotellaceae	0.1543	0.2147	0.1855	0.1367
Veillonellaceae	0.1141	0.0848	0.0647	0.0646
Bacteroidaceae	0.1756	0.2078	0.1608	0.2213^∗^
Bifidobacteriaceae	0.0076	0.0225	0.0105	0.0305
Ruminococcaceae	0.1995	0.1608	0.2178	0.1853
Lachnospiraceae	0.1781	0.1488^∗^	0.1639	0.1435
Enterobacteriaceae	0.0220	0.0352	0.0348	0.0648
Porphyromonadaceae	0.0217	0.0256	0.0328	0.0327
Christensenellaceae	0.0123	0.0065	0.0176	0.0152
Erysipelotrichaceae	0.0090	0.0034^∗^	0.0047	0.0032^∗^
Rikenellaceae	0.0142	0.0148	0.0216	0.0235
Bacteroidales_S24_7_group	0.0056	0.0061	0.0010	0.0035
Streptococcaceae	0.0062	0.0079	0.0101	0.0097
Lachnospiraceae_1	0.0085	0.0029^∗^	0.0065	0.0054
Verrucomicrobiaceae	0.0170	0.0164	0.0154	0.0144
Clostridiaceae_1	0.0006	0.0003	0.0032	0.0026
Desulfovibrionaceae	0.0078	0.0047^∗^	0.0062	0.0033
Alcaligenaceae	0.0050	0.0061	0.0069	0.0060^∗^
Acidaminococcaceae	0.0061	0.0064	0.0079	0.0071
Peptostreptococcaceae	0.0074	0.0064	0.0046	0.0073^∗^
Methanobacteriaceae	0.0091	0.0046^∗^	0.0075	0.0040^∗^
Synergistaceae	0.0004	0.0000^∗^	0.0005	0.0017
Bacillaceae	0.0002	0.0001	0.0002	0.0001^∗^
Elusimicrobiaceae	0.0007	0.0004^∗^	0.0006	0.0005
Clostridiales_vadinBB60_group	0.0003	0.0001^∗^	0.0003	0.0003

*^∗^Significant difference between the two groups, P < 0.05.*

### Variation Analysis of Gut Microbiome Associated With Rosuvastatin Treatment

**FIGURE 4:**
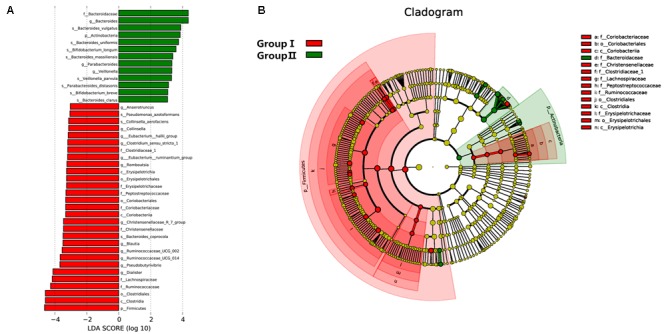
Distribution of microbiome was compared between two groups by using linear discriminant analysis (LDA) effect size (LEfSe). **(A)** Histogram of the LDA scores reveals the most differentially abundant taxa in two groups. The taxa listed in red represent group I and green describe group II. **(B)** The cladogram shows the significantly overrepresented bacterial taxa in group I (red area) and group II (green area).

**FIGURE 5:**
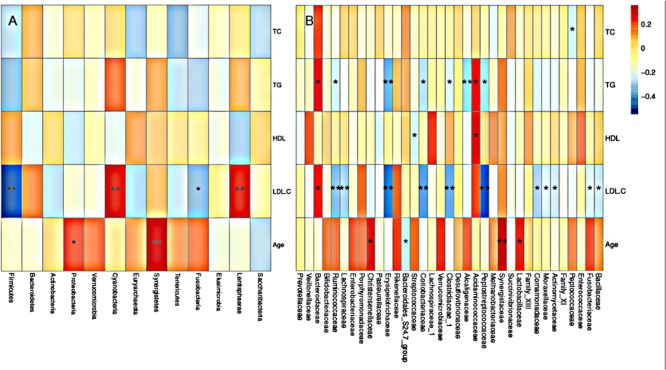
Gut microbiota according to serum lipid level and age. Heatmaps of Spearman correlations display the relationships between bacterial phyla **(A)**, or bacterial family **(B)** and blood lipids or age. Gut microbiota according to serum lipid level and age.

To further assess the correlation between gut microbial taxa, rosuvastatin effect and age, LDA coupled with effect size measurements (LEfSe) was applied to identify the differentially abundant OTUs between group I and II. A total of 42 taxa manifested a significant difference in their abundance between the two groups (LDA score > 3.0). 29 microbial taxa were enriched in group I compared to group II, including 1 bacterial phylum, 3 classes, 3 order, 7 families, 12 genera, and 3 species. Group II was characterized by higher abundance of other 13 discriminatory taxa, including 1 phylum, 1 family, 3 genera, and 8 species. These taxa were depicted in **Figure 4**. Spearman’s rank correlation method was used for analyzed the associations of variation of fecal microbiome with serum lipids and age (**Figure 5**). Among differentially abundant bacterial phyla, Firmicutes or Fusobacteria were correlated negatively with LDL-C (*P* < 0.05). The positive correlation were found between LDL-C and Cyanobacteria and Lentisphaerae, as well as between the age and Proteobacteria and Synergistetes (*P* < 0.05). At the family level, TG and LDL-C were positively correlated with Bacteroidaceae or Acidaminococcaceae (*P* < 0.05) and negatively correlated with Lachnospiraceae, Ruminococcaceae, Erysipelotrichaceae, Coriobacteriaceae, Clostridiaceae_1, Alcaligenaceae, Peptostreptococcaceae, Moraxellaceae, Actinomycetaceae, or Fusobacteriaceae (*P* < 0.05). Moreover, the age was positively associated with Lactobacillaceae, Christensenellaceae, or Synergistaceae, and negatively associated with Bacteroidales_S24.7_group (*P* < 0.05).

## Discussion

The present study explored the effects of rosuvastatin lowering lipid levels in the hyperlipidemia patients. After 4–8 weeks therapy, different therapeutic efficacy was observed in the participants. The patients were then divided into two parallel groups, group I reached an optimal levels of TC, TG, and LDL-C following rosuvastatin treatment, group II also achieved a lipid-lowering effect but without reaching the normal. Rosuvastatin is an effective lipid-lowering drug, which is commonly used for the treatment of hyperlipidemia on the doses of 10 mg per day in China (Ding et al., 2015). However, although the efficacy of rosuvastatin having been well established, many patients did not achieve ideal effects. Karlson et al. (2016) assessed mean percentage change in LDL-C among 38,052 subjects with rosuvastatin oral tablet 5–40 mg daily. From the results with VOYAGER meta-analysis they observed that rosuvastatin 5 mg decreased LDL-C by 39% and non-HDL-C by 35%, while rosuvastatin 40 mg reduced LDL-C by 55% and non-HDL-C by 50% in patients with hyperlipidemia. Anagnostis et al. (2014) reported that 12-week-treatment period of rosuvastatin with the doses of 10 mg daily led to a 49.4% reduction in LDL-C, a 37% decrease in TC and a 27.4% decline in TG in non-diabetic patients with dyslipidemia. These findings demonstrate that increasing the dose of rosuvastatin and prolonging the time of drug administration lead only to a limited reduction in the serum lipids levels. Our results were in agreement with these findings reported in the literature.

The human gastrointestinal microbiota act as not only an important barrier to aggressive agents but also as modulator in maintaining symbiotic homeostasis and normal physiology (Robles-Alonso and Guarner, 2013; Goulet, 2015). In recent years, a growing body of literature uncover that imbalance of gut microbiota are closely related to the occurrence and development of a number of diseases, including obesity, diabetes, cardiovascular disease, allergies, cancer, and so on (Ley et al., 2006). Due to the development of 16S rRNA sequencing technologies, variations of human gut microbiome that associate with dyslipidemia have been well documented by researchers. Fu et al. (2015) analyzed the relevance between the human intestinal bacteria taxa and blood lipid levels in 893 subjects. They found 34 bacterial taxa were significantly correlated with blood lipids levels, accounting for 6% of the variance in TC, and 4% in HDL, independent of other influence factors (Fu et al., 2015). A study investigating anti-hyperlipidemia of RC alkaloids revealed that RC alkaloids were able to change the composition of gut microbiota and restore the balance of microflora, hence leading to a lipid-lowering effect (He et al., 2016). These findings indicate that gut microbiota may play crucial role in etiology of dyslipidemia. The underlying mechanisms of gut microflora affects blood lipid levels remain unclear. It has been addressed that gut bacteria generate SCFAs which can modulate hepatic and/or systemic lipid and glucose metabolism via the activation of nuclear or G protein-coupled receptors (GPCRs). As well, gut microflora can modulate the metabolism of bile acids that have been implicated in the regulation of lipid, glucose, and energy metabolism.

High-throughput DNA sequencing of 16S rRNA genes isolated from stool specimens was used to produce large numbers of valid reads for further microbiome analysis in this study. We showed that the compositions of gut microbiota were different between subjects with hyperlipidemia who displayed optimal (group I) or suboptimal (group II) clinical outcomes following rosuvastatin intervention. Alpha diversity analysis demonstrated a higher complexity of fecal microbiome than that of group II. In particular, we identified that higher abundances of phylum of Firmicutes, BPB families of Ruminococcaceae, Lachnospiraceae, Clostridiaceae-1, and lower abundances of phylum of Bacteroidetes were associated with an optimal therapeutic effect of rosuvastatin. Firmicutes participate in the metabolic process of phenolic compounds, which act as antidiabetic and anti-obesity agents (Selma et al., 2009; Eid et al., 2017). Thus this bacterial phylum was indicated a potential role in the maintenance of normal blood lipids. BPB are located in the colon and distal small intestine, which can ferment dietary fiber into a SCFAs named butyrate. Decreased numbers of BPB has been involved in a number of disorders such as fatty liver disease, inflammatory bowel diseases, diabetes, and so on (Willing et al., 2010; Qin et al., 2012; Endo et al., 2013). Weng et al. (2015) revealed that butyrate-producing probiotics along with fiber administration reduced adipose tissue mass and serum TG level in mice. This finding indicated that BPB may act as a new therapeutic target against hyperlipidemia and obesity. Lu et al. (2017) observed an elevated ratio of Firmicutes to Bacteroidetes and decreased abundance of probiotics in mice fed a high-sugar-high-fat diet. We found group I exhibited an increased abundances of probiotics such as Lactobacillaceae and Bifidobacteriaceae and achieved higher effectiveness of rosuvastatin treatment. Researchers have explored the therapeutic potential and the underlying mechanisms of probiotics for dyslipidemia in recent years. For example, administration of Lactobacillus in rats fed with a high-lipid diet contributed to a significant reduction in serum TC, TG, and LDL, and significantly increased HDL (Wang et al., 2012). Prebiotic feeding up-regulated the concentrations of plasma gut peptide (glucagon-like peptide 1 and peptide YY), leading to alterations in appetite sensation and the lowered hunger ratings in healthy subjects (Cani et al., 2009). In another report, two probiotic strains, *Lactobacillus plantarum* KY1032 and *L. curvatus* HY7601, were shown to improve the hepatic mRNA expression of PPARα, bile acid receptor, and plasma apolipoprotein A–V in hypertriglyceridemic rats, thus exerting lipid-lowering effect (Choi et al., 2016).

Opposite to the role of probiotics in maintaining lipid homeostasis, certain types of bacteria inhabiting the intestines had the potential to induce fat accumulation and obesity, this could be confirmed by the work from Bäckhed F’s team. They compared germ-free and colonized mice supplied with high-fat and high-sugar diet, and found germ-free animals were protected from the obesity by elevating Fiaf and enhancing the activity of phosphorylated AMP-activated protein kinase in muscle and liver (Backhed et al., 2007). High-fat diet feeding was involved in changed compositions of gut microbes and increased plasma lipopolysaccharide (endotoxin of Gram-negative bacteria) levels (Cani and Delzenne, 2009). Bacterial endotoxin has been reported to cause adipose tissue formation, systemic and local inflammatory and metabolic disorders, which were defined “metabolic endotoxemia” (Boutagy et al., 2016). Our results noticed a higher richness of Actinobacteria, Lentisphaerae, Bacteroidaceae, Prevotellaceae, Pseudomonadaceae, Streptococcaceae, Acidaminococcaceae, Enterobacteriaceae, Citrobacter, Veillonella, and Escherichia_Shigella in group II, most of these bacteria belong to endotoxin-producing microbes. Their high levels may explain partially the reason why rosuvastatin was not that effective in subject communities from group II. Generally, how the gut microflora impact on the metabolism and pharmacokinetics of statins is unknown. This might involve the microbiota together with the host cells to maintain intestinal homeostasis, to change statin-metabolizing enzymes and transporters and to modulate the activity of drug receptors.

In addition, we did not observe significant difference in microbial diversity between two groups. However, the women cohort in group I showed higher richness of Firmicutes, Ruminococcaceae, Lachnospiraceae, Peptostreptococcaceae, Family_XIII bacteria, Defluviitaleaceae bacteria and lactobacillus, as well as lower abundance of Saccharibacteria, Bacteroidaceae, Prevotellaceae, and Enterobacteriaceae as compared to female in group II. For male subjects in group I, markedly elevation in bacterial phylum of Firmicues, families of Peptostreptococcaceae and Defluviitaleaceae was observed in comparison to those in group II. The data suggested that gender should be considered as an influence factor together with gut microbiota compositions in rosuvastatin treatment. Comparison between subjects ranging from 55–65 to 66–75 in two groups revealed that distribution of BPB was different based on age. Age-related variation of the microbiota occurs as an ecological succession under the multiple perturbations associated with the conditions as chronic gastrointestinal diseases, metabolic disorders antibiotic use (Lozupone et al., 2012). Our age associated species Lactobacillaceae, Christensenellaceae, or Synergistaceae did not apply to the usual situation. However, the gut flora of individual subjects may display little divergence with small age difference.

We also observed that the association was present between rosuvastatin efficiency and the microbial diversity of human gut. As the TG and LDL-C levels changed, the diversity of the composition of gut microbiome was spotted, but not the richness. Overall, the patient gut microbiota exhibited variation in community diversity and taxon abundance in association to rosuvastatin hypolipidemic effect. These results indicate that gut microbiota have potency in influencing statin treatment to patients with dyslipidemia; the modulation of gut microflora, especially statin effectiveness correlated species, might provide a management for treating statin-inadequate patients. 16S rRNA sequencing technique gives a lot of information on the abundance and diversity of microflora. There is scarce knowledge of their functions and underlying mechanisms involved in aberrant blood lipids, which we hope learn more in the future.

## Availability of Data and Materials

The datasets analyzed during the current study are available in the SRA NCBI repository under the Bioproject accession number PRJNA414024.

## Author Contributions

YL contributed to the study design, experiment performing, and the manuscript. XS and HZ contributed to result interpretation, data processing and analysis, and manuscript writing. XZ and SW contributed to the data analyses. YX contributed to the sample collection. XD, WZ, ST, and LW performed some experiments. JX and LT contributed to the study design and data analyses. All authors contributed to critical revisions to the manuscript and approved the final version for submission.

## Conflict of Interest Statement

The authors declare that the research was conducted in the absence of any commercial or financial relationships that could be construed as a potential conflict of interest.

## Footnotes

1 http://ccb.jhu.edu/software/FLASH/

2 http://qiime.org/scripts/split_libraries_fastq.html

3 http://www.drive5.com/usearch/manual/uchime_algo.html

4 http://drive5.com/uchime/uchime_download.html

5 www.r-project.org

## Abbreviations

ACE: abundance-based coverage estimators
AEs: adverse events
ANOSIM: analysis of similarities
BPB: butyrate-producing bacteria
FDR: false discovery rate
Fiaf: fasting-induced adipose factor
HDL: high density lipoprotein
LDA: linear discriminate analysis
LDL-C: low-density lipoprotein cholesterol
LefSe: linear discriminant analysis effect size
OTUs: operational taxonomic units
PCA: principal component analysis
RC: Rhizoma Coptidis
SCFA: short-chain fatty acid
TC: total cholesterol
TG: triglycerides
VLDL: very low density lipoprotein
VLDL-C: very low density lipoprotein cholesterol
